# Maternal and neonatal outcomes of grand multiparity in Khartoum, Sudan

**DOI:** 10.4314/ahs.v22i1.21

**Published:** 2022-03

**Authors:** Elmugabil Abdelmageed, Hassan Bahaeldin, Alhabrdi Nadiah, Ahmed Abdelbagi, Rayis Duria, Adam Ishag

**Affiliations:** 1 University of Khartoum Faculty of Medicine, obstetrics and gynecology; 2 Department of obstetrics and gynecology, College of Medicine, King Khalid University, Abha, Saudi Arabia; 3 Department of Obstetrics and Gynecology, Unaizah College of Medicine and Medical Sciences, Qassim University, Unaizah, Kingdom of Saudi Arabia; 4 Department of obstetrics and gynecology, College of Medicine, King Khalid University, Abha, Saudi Arabia; 5 University of Khartoum Faculty of Medicine, Obstetrics, and Gynecology; 6 Department of Obstetrics and Gynecology, Unaizah College of Medicine and Medical Sciences, Qassim University, Unaizah

**Keywords:** Grand multiparity, multiparous, maternal outcome, neonatal outcome, Sudan

## Abstract

**Objectives:**

Grand multiparity is a major health problem that leads to adverse maternal and perinatal outcomes. We aimed to assess the maternal and perinatal outcomes of grand multiparity.

**Methods:**

A case-control study was conducted in Saad Abualila Hospital, Khartoum, Sudan from February to December 2019. The cases were grand multiparous (≥ 5 deliveries) women. The controls were women with low parity (multiparous women who delivered two to four times). Maternal and perinatal characteristics were compared between the two groups. Logistic regression analysis was performed.

**Results:**

There was a significant association between grand multiparity and higher maternal age (adjusted odds ratio [AOR]=1.19, 95% confidence interval [CI]=1.16–1.23), lower education level (AOR=3.38, 95% CI=2.49–5.58) and lower antenatal care attendance (AOR=1.73, 95% CI=1.02–2.92). Grand multiparous women were at increased risk for Anemia (AOR=1.48, 95% CI=1.08–2.03), diabetes mellitus (AOR=10.61, 95% CI=4.89–23.00), caesarean delivery (AOR=1.87, 95% CI=1.40–2.48), preterm birth (AOR=1.90, 95% CI=1.37–2.62) and admission to the neonatal intensive care unit (AOR=3.8, 95% CI=1.95–7.75).

**Conclusions:**

Grand multiparity was associated with poor maternal and neonatal outcomes. Development of a national health program addressing family planning, health education and improvement of antenatal, intrapartum and neonatal care are needed.

## Introduction

Grand multiparity, which is defined as five or more deliveries, is a major obstetric problem in developing countries^1^. Unlike the low prevalence (3–4%) of grand multiparity in developed countries^2^, developing countries, especially in Sub-Saharan Africa, have a high prevalence of grand multiparity, which varies between 17% and 33 %^2^,^3^. In Africa, grand multi parity has been attributed to factors such as poverty, unavailability of health care resources, poor antenatal care, illiteracy and shortage of modern contraceptive practices.

The determinants of grand multiparity in previous studies were a desire for more children, unplanned pregnancy, less intention to use contraceptives and death of another child^3^,^5^. Moreover, marriage at an early age in developing countries also contributes to high parity.

Grand multiparity can lead to poor maternal and fetal outcomes, such as antepartum and post-partum hemorrhage, gestational diabetes, hypertension, Anemia and preterm birth^2^,^7^. Perinatal complications associated with grand multiparity include fetal malpresentation, cephalopelvic disproportion, congenital malformation, fetal macrosomia, increased intensive care unit admission and perinatal mortality.

To improve maternal and fetal outcomes in developing countries, efforts should be directed towards reduction of the incidence of high parity. Some strategic plans to achieve reduction in the incidence of grand multiparity have been enacted; these include health education, empowerment of antenatal care facilities, encouragement of first marriage at a late age and community awareness and practice of modern contraceptives

[In Sudan, maternal and perinatal mortality are amongst the highest in the region and the world^9^,^10^. However, the epidemiology and effects of grand multiparity have not been fully documented in Sudan. The current study was conducted at a tertiary hospital in Khartoum, Sudan, from February to December 2019 to determine the maternal and perinatal outcomes of grand multiparity.

## Methods and Materials

A retrospective case-control study was conducted at Saad Abuelela Tertiary Hospital in Khartoum, Sudan from February to December 2019. Ethical approval was received from the Ethics Committee at the Department of Obstetrics and Gynaecology, Faculty of Medicine, University of Khartoum, Sudan (reference number: 2018/09). Written informed consent was collected from each participant before taking part in the research. The cases were women who had delivered five or more times (grand multiparae). Mothers who had delivered two to four times (multiparae) were the controls. All the women in the study gave birth to a single infant. Primiparae, seriously ill mothers, twin births and congenital malformed deliveries were excluded.

All mothers signed informed consent form. Trained medical officers gathered the socio-demographic, obstetric, and perinatal information.

For each woman, age, parity, education, occupation, level of antenatal care attendance, gestational age, interpregnancy interval (IPI), early pregnancy (<14 weeks), body mass index (BMI), haemoglobin level, birth weight and infant's sex were recorded. The investigators were not involved in the management or care of the mothers or the newborns. The main outcome measures (maternal and perinatal outcomes) were recorded from clinical notes and included pregnancy complications, such hypertension, preeclampsia or diabetes (gestational or chronic), hemorrhage, mode of delivery, admission to the neonatal intensive care unit and perinatal death.

Early pregnancy (<14 weeks) weight and height were used to calculate BMI as weight in kilograms divided by the squared height in meters. The World Health Organization classification was used to classify the women according to their BMI: normal weight (18.5–24.9 kg/m^2^), overweight (25.0–29.9 kg/m2) or obese (30.0–34.9 kg/m^2^) 11.

Haemoglobin was an automated hematology analyzer, according to the manufacturer's instructions (Sysmex KX-21, Japan).

Women who had more than five deliveries is defined as grand multipara^12^. Hypertensive disorders of pregnancy were defined as blood pressure > 140/90 mmHg noted on two or more occasions after 20 weeks of gestation in a previously normotensive woman with or without proteinuria^13^. Preterm delivery was defined as birth occurring before 37 weeks of gestation^14^. Anemia was defined as haemoglobin <11.0 g/d^15^.Perinatal death was defined as a newborn delivered after 28 weeks of gestation who either showed no signs of life (stillbirth) or who was delivered alive and died within one week of life. Mothers who were discharged from the hospital were followed up with a telephone call to inquire about their newborn^16^.

The sample size was calculated guided by the prevalence of grand multiparity in nearby countries (20.0%)^17^ and a case: control ratio of 1:2. The required sample size was determined considering a prevalence of anemia of 50% in the cases and 40% in the controls (parous). This sample size would have a type I error of 5% and adequate power (80% of power, β = 0.2). The final sample included 362 cases and 724 controls, taking into account that 10% of the women might not respond or might provide incomplete data.

### Statistics

Data were entered into a computer, and SPSS version 18 for Windows (SPSS Inc., Chicago, IL, USA) was used for data analysis. Continuous data were checked for normality using the Shapiro-Wilk test. The mean (standard deviation), median (interquartile range), frequency and percentage were used to present the participants' characteristics. Maternal and perinatal characteristics were compared using the Student-t, Mann-Whitney, X2 and Fisher exact tests where applicable.

Logistic regression with grand multiparity as the dependent factor and the medical and obstetric factors, including the mother's age, parity, education, care attendance, history of previous miscarriages/preterm birth, haemoglobin level and infant sex, as independent factors. were performed for the maternal and perinatal outcomes with dependent and independent variables as shown in Table 1. Variables with a p-value of 0.20 were logistic regression model using the backward stepwise crude odds ratio, adjusted odds ratio (AOR) and 95% to show the strength of the association. A two-sided statistically significant.

**Table 1:** Dependent and independent variables for determinants, maternal and perinatal outcomes of multipara

Independent variables	Dependent variables
Age, education, antenatal care, occupation, history of miscarriage/ preterm birth, interpregnancy interval, body mass index,	Grandmultipra
Age, education, antenatal care, occupation, history of miscarriage/ preterm birth, interpregnancy interval, body mass index and parity	Anemia
Age, education, antenatal care, occupation, history of miscarriage/ preterm birth interpregnancy interval, body mass index, parity, anemia	Preterm birth
Age, education, antenatal care, occupation, history of miscarriage/ preterm birth, interpregnancy interval, body mass index, parity, anemia, gestational age	Caesarean delivery
Age, education, antenatal care, occupation, history of miscarriage/ preterm birth, interpregnancy interval, body mass index, parity, anemia, gestational age	Hypertensive disorder
Age, education, antenatal care, occupation, history of miscarriage/ preterm birth, interpregnancy interval, body mass index, parity, anemia, gestational age	Diabetes mellitus
Age, education, antenatal care, occupation, history of miscarriage/ preterm birth, interpregnancy interval, body mass index, parity, anemia, gestational age, gender	Admission to neonatal intensive care unit

## Results

**Figure 1:**
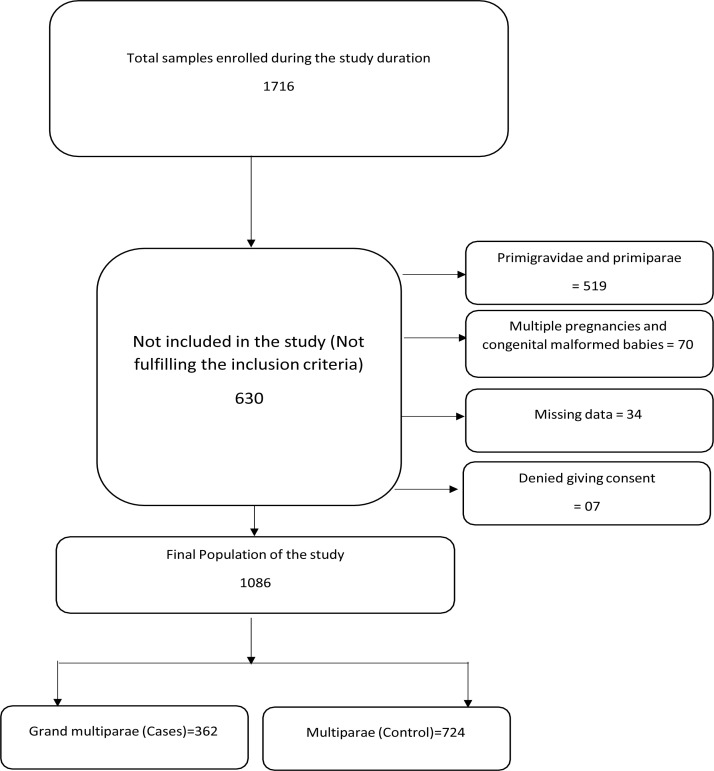
The Study Flow Chart.

During the study period, there were 1716 mothers recruited ,630 were excluded due to parity status(519), multiple pregnancy and congenital malformation(70), missed data (34) and denial to give consent(7), The final sample included 362 grand multiparae cases (21.09%) and 724 multiparae as controls (figure 1).

The median (interquartile range) of the age and IPI of grandmultiparae were significantly higher than in the parous mothers. Compared to the parous mothers, grand multiparae had a significantly higher history of miscarriage and lower levels of education and antenatal care attendance.

Details of the comparison of sociodemographic characteristics between the two groups are shown in Table 2.

**Table 2:** Comparing sociodemographic and clinical variables between grand multiparas and parous women

Variables	Grand mulitparas (362)	Mulitparas (724)	OR (95%CI)	P
Age, years	33.0(30.0 .36.0)	28.0(25.0–32.0)	1.16(1.13–1.19)	<0.001
Interpregnancy interval, (months)	25.0(18.0–36.0)	23.0(15.0–32.0)	1.01(1.01–1.03)	0.005
Miscarriage/preterm birth				
Yes	120(33.1)	201(27.8)	1.29(0.98–1.69)	0.078
No	242(66.9)	523(72.2)	Reference	
Occupation				
Housewife	326(90.1)	638(88.1)	Reference	
Employee	36(9.9)	86(11.9)	1.22(0.80–1.84)	0.361
Antenatal care				
. two visits	318(87.8)	682(94.2)	Reference	
. two visits	44(12.2)	42(5.8)	2.24(1.44–3.50)	<0.001
Body mass index(kg/m2)				
Underweight	2(0.6)	10(1.4)	0.35(0.07–1.64)	0.187
Normal weight	156(43.1)	278(38.4)	Reference	
Overweight	140(38.7)	312(43.1)	0.80(0.60–1.05)	0.117
Obese	64(17.7)	124(17.1)	0.92(0.64–1.31	0.649
Education level				
. secondary level	136(37.6)	430(59.4)	Reference	
< secondary level	226(62.4)	294(40.6)	0.55(0.46–0.65)	<0.001

Compared to parous mothers, grand multiparae had a significantly higher prevalence of Anemia, preterm birth, caesarean delivery, admission to the neonatal intensive care unit and perinatal mortality (see Table 3).

**Table 3:** Comparing maternal and perinatal outcomes between multiparae and parous women

Variables	Grand mulitparas (362)	Mulitparas (724)	OR (95%CI)	P
Anemia				
Yes	263(72.7)	463(64.0)	1.49(1.13–1.97)	
No	99(27.3)	261(36.0)	Reference	0.004
Hypertensive disorder				
Yes	29 (8.0)	32(4.4)	1.46(1.10–1.93)	
No	333(92.0)	692(95.6)	Reference	0.018
Diabetes mellitus				
Yes	34 (9.4)	9(1.2)	2.51(2.10–3.00)	
No	328(90.6)	715(98.8)	Reference	<0.001
Antepartum haemorrhage				
Yes	8 (2.2)	5(0.7)	1.86(1.20–1.89)	
No	354(97.8)	719(99.3)	Reference	0.039
Delivery				
Caesarean	213 (58.8)	342(47.2)	1.59(1.23–2.06)	
Vaginal	149(41.2)	382(52.8)	Reference	<0.001
Preterm birth				
Yes	87(24.0)	114(15.7)	1.69(1.23–2.31)	
No	275(76.0)	610(84.3)	Reference	0.001
Gender				
Female	180(49.7)	359(49.6)	Reference	
Male	182(50.3)	365(50.4)	0.99(0.84–1.17)	0.966
Admission to neonatal intensive care unit				
Yes	24(6.6)	21(2.9)	2.37(1.30–4.33)	
No	338(93.4)	703(97.1)	Reference	0.004
Perinatal mortality				
Yes	9 (2.5)	14(1.9)	1.29(0.55–3.01)	
No	353 (97.5)	710(98.1)	Reference	0.655

In the multivariate logistic regression analysis, higher age (AOR=1.19, 95% CI=1.16–1.23, P<0.00), lower education age (AOR=3.38, 95% CI=2.49–5.58, P<0.00) and lower antenatal care attendance (AOR=1.73, 95% CI=1.02–2.92, P=0.04) were significantly associated with grand multiparity, see (Table 4).

**Table 4:** Logistic regressions of sociodemographic and clinical variables associated with grandmultipara

	Unadjusted		Adjusted	
Variables	OR (95%CI)	P	AOR (95%CI)	P
Age, years	1.19(1.16–1.23)	<0.001	1.19(1.16–1.23)	<0.001
Interpregnancy interval	0.98(0.98–1.01)	0.688	0.99(0.098–1.01)	0.676
Miscarriage/preterm birth				
Yes	1.13(0.82–1.54)	0.436	1.12(0.82–1.153)	0.462
No	Reference		Reference	
Education level				
≥ secondary level	Reference			
< secondary level	3.36(2.47–4.57)	<0.001	3.38(2.49–4.58)	<0.001
Antenatal care				
≥ two visits	Reference			
< two visits	1.75(1.03–2.97)	0.038	1.73(1.02–2.92)	0.040
Body mass index(kg/m2)				
Underweight	0.43(0.08–2.28)	0.325	0.85(.239–3.073)	0.813
Normal weight	Reference		Reference	
Overweight	0.84(0.61–1.15)	0.286	0.880(.649–1.193)	0.409
Obese	0.83(0.55–1.24)	0.368	1.133(.753–1.706)	0.548

Grand multiparity was significantly associated with Anemia (AOR=1.48, 95% CI=1.08–2.03, P=0.5, diabetes mellitus (AOR=10.61, 95% CI=4.89–23.00, P<0.01), caesarean delivery (AOR=1.87, 95% CI=1.40–2.48, P<0.001), preterm birth (AOR=1.90, 95% CI=1.37–2.62, P<0.001) and admission to neonatal intensive care unit unit (AOR=3.80,95% CI=1.95–7.75, P<0.001); Table 5.

**Table 5:** Logistic regressions of associations between grandmultipara with maternal and perinatal outcomes

	Unadjusted		Adjusted	
Variables	OR (95%CI)	P	AOR (95%CI)	P
Anemia	1.47(1.07–2.02)	0.016	1.48(1.08–2.03)	0.015
Hypertensive disorder	1.77(0.98–3.18)	0.055	1.63(0.93–2.86)	0.088
Diabetes mellitus	12.00(5.19.27.75)	<0.001	10.61(4.89.23.00)	<0.001
Caesarean Delivery	1.93(1.44–2.95)	<0.001	1.87(1.40–2.48)	<0.001
Preterm birth	1.68(1.16–2.42)	0.005	1.90(1.37–2.62)	<0.001
Admission to neonatal intensive care unit	4.34(2.12–8.8)	<0.001	3.8(1.95–7.75)	<0.001

## Discussion

Amongst 1716 deliveries during the study period, there were 362 grand multiparous women (21.09%). This prevalence is not out of the range of prevalence of grand multiparous women in other countries in the sub-Saharan zone (17–33%), e.g. Gambia (26.5%)^3^ and Cameroon (27.0%)^17^. A much lower prevalence (9.44%) of grand multiparous women has been reported in Tanzania^2^. Moreover, in neighboring Saudi Arabia, which might be thought to have the same tradition, the prevalence (10.2%) of grand multiparous women is much lower than we observed for Sudan^7^.

The difference in the prevalence of grand multiparous women could be explained by differences in traditions and contraceptive use. In the current study, a higher age (AOR=1.19) was associated with grand multiparity. The mean age of 33 years in our study is similar to the age reported in Cameroon^1^. A mean age of 33 years for grand multiparous women means they may have more pregnancies, which alerts us to the need to encourage birth control methods in our community to decrease adverse pregnancies. A low level of education was associated with grand multiparity in our study. Similar observations of less education amongst grand multiparous women have been reported in other studies^8^,^18^. In Sudan, early marriage might obligate mothers to leave school, especially in rural areas, and hence early marriage leads to both grand multiparity and low education. The association between grand multiparity and low levels of attendance for antenatal care could be because of time management, limited resources in the family and negative perceptions resulting from previous pregnancies. It is also possible that multiparous mothers, who have greater experience, feel more confident during pregnancy and consider antenatal care less important.

Gravidity may be associated with micronutrient deficiency status in pregnant women^22^. It is postulated that pregnant women with high parity (repeated pregnancies) have low or depleted iron storage [23]. Moreover, in our study, both diabetes mellitus (AOR=10.61) and caesarean delivery (AOR=1.87) were associated with grand multiparity. Our results agree with the results of previous studies^24^,^25^. Increased incidence of caesarean delivery amongst the grand multiparous women was probably due to malpresentation, which is common complication in this group of women^26^.

In the current study, grand multipara were 1.48 times (AOR=1.48) more likely to have Anemia. We reported in a meta-analysis that the pooled prevalence of Anemia amongst pregnant women in Sudan was 53.0%, and Anemia was not associated with parity^19^. Similar results have been reported in a meta-analysis in Ethiopia, which found that primigravidae were at lower risk of having Anemia^20^. Moreover, pregnant women with gravidity six and above were at a 2.59 higher risk for Anemia^21^.

Conversely, in Saudi Arabia, grand multipara is less likely to have caesarean delivery (odds ratio: 0.60, 95% CI: 0.40–0.80^7^.

In the present study, in accordance with previous studies, grand multiparae had a significantly higher prevalence of preterm birth^2^,^7^. Previously published studies have reported that in the in the presence of regular antenatal care and adequate intrapartum care grand multiparity is not associated with a significantly increased risk of these obstetric complications^1^,^7^. An increased number of admissions to the neonatal intensive care unit in the grand multiparous group was observed when compared with the multiparous women group (AOR=3.80). Previous studies have reported similar findings.^6^,^7^.

In the current study, perinatal mortality was significantly higher amongst the grand multiparous group. Similar findings have been reported in previous studies^2^,^26^. In contrast to our perinatal outcome data, two Saudi and Sudanese studies reported no association between grandmultiparae and poor perinatal outcomes^27^,^28^. Interestingly, one study in Saudi Arabia, neither maternal nor perinatal adverse effects were associated with grandmultiparae^7^, modern prenatal and neonatal care in Saudi Arabia are possible explanations for this observation.

## Conclusion

This study indicated a prevalence of grand multiparity (21.09%) that is comparable to the prevalence (17%–33%) of multiparity in the sub-Saharan region. Grand multiparity in this study was associated with low education, fewer antenatal care visits and adverse maternal and neonatal outcomes.

To improve maternal and neonatal outcomes in Sudan, we need to ring the bell for the reduction of the prevalence of grand multiparous women in the community develop a national health program to reduce the prevalence of grand multiparity.

The program pillars should include family planning and health education of the risk of grand multiparity and the importance of antenatal
